# Hydrological connectivity promotes coalescence of bacterial communities in a floodplain

**DOI:** 10.3389/fmicb.2022.971437

**Published:** 2022-09-21

**Authors:** Baozhu Pan, Xinyuan Liu, Qiuwen Chen, He Sun, Xiaohui Zhao, Zhenyu Huang

**Affiliations:** ^1^State Key Laboratory of Eco-hydraulic in Northwest Arid Region of China, Xi'an University of Technology, Xi'an, Shaanxi, China; ^2^State Key Laboratory of Hydrology-Water Resources and Hydraulic Engineering, Nanjing Hydraulic Research Institute, Nanjing, China; ^3^State Key Laboratory of Crop Stress Biology in Arid Areas, Shaanxi Key Laboratory of Agricultural and Environmental Microbiology, College of Life Sciences, Northwest A&F University, Yangling, Shaanxi, China

**Keywords:** community coalescence, co-occurrence network, hydrologic connectivity, river floodplain, planktonic bacteria, sedimentary bacteria

## Abstract

Floodplains play essential roles in the ecological functions of regional environments. The merging and coalescence of bacterial communities in aquatic environments results in periodic patterns driven by regular hydrological activities, which may, in turn, influence ecological activities. However, the degree of bacterial community coalescence in the lateral and vertical directions as well as the underlying hydrological mechanism of floodplain ecosystems is poorly understood. Therefore, we investigated the spatiotemporal patterns and coalescence processes of planktonic and sedimentary bacterial communities during normal and high-water periods in a floodplain ecosystem of the Yellow River source region. We classified bacterial operational taxonomic units (OTUs) based on 16S rRNA gene sequencing, and quantified community coalescence by calculating the proportions of overlapping OTUs, the contributions of upstream sources to downstream sinks, and positive/negative cohesion. The results revealed major differences in the composition and diversity of planktonic and sedimentary bacterial communities. Bacterial community diversity in the high-water period was higher than in the normal period. Laterally, hydrological connectivity promoted the immigration and coalescence of bacterial communities to oxbow lakes in both the mainstream and tributaries, with the coalescence degree of planktonic bacteria (2.9%) higher than that of sedimentary bacteria (1.7%). Vertically, the coalescence degree of mainstream planktonic and sedimentary bacterial communities was highest, reaching 2.9%. Co-occurrence network analysis revealed that hydrological connectivity increased the complexity of the bacterial network and enhanced the coalescence of keystone species to oxbow lakes. Furthermore, community coalescence improved the competitiveness and dispersal of bacterial communities. This study demonstrated that coalescence of bacterial communities is driven by hydrological connectivity in a floodplain ecosystem. Further studies should investigate the processes of bacterial community coalescence in floodplains in more detail, which could provide new approaches for environmental protection and ecological function preservation.

## Introduction

Floodplains are alluvial complexes comprising interconnected biota and ecological gradients. Being extremely vital ecosystems (Argiroff et al., 2017; Wang et al., 2020), floodplains contribute to preserving biodiversity (Naiman et al., 1993), maintaining water quality (Mitsch et al., 2001; Tockner et al., 2010) and handling flood surges (Kousky and Walls, 2014). Floodplains contain a vertical tree-like network of mainstream and tributaries (Mansour et al., 2018). In addition, numerous ecological niches are present as oxbow lakes, formed by shore erosion and overflow floods, which are seasonally separated from the original rivers (Durkin et al., 2015; Wang et al., 2020). During the normal period, oxbow lakes are partitioned from the mainstream, exhibiting a high spatial heterogeneity (Mayora et al., 2020). However, during the high-water period, the rising water of the mainstream will flood the floodplain between the mainstream and the oxbow lake, and the mainstream and the oxbow lake are connected. As a result, most environment of mainstream and oxbow lake displays typical equilibrium effects (Mayora et al., 2013, 2020). Hydrological connectivity not only drives matter and energy flows laterally and vertically, but also maintains the spatiotemporal heterogeneity of microbial community structure in riverine networks (Mansour et al., 2018).

Bacteria constitute a substantial part of microbial communities and play a paramount role in biogeochemical processes and nutrient cycling in aquatic ecosystems (Findlay, 2010; Madsen, 2011; Zhang et al., 2022a,b). According to their habitat preferences in rivers and lakes, bacterial communities can be divided into planktonic and sedimentary. The planktonic bacterial community is the sum of the sources of upstream bacteria, including rainfall, lake water, groundwater, and soil water, and it is susceptible to compositional and structural variations (Liu et al., 2018). The sedimentary bacterial community is formed through long-term sediment erosion and accumulation (Qian et al., 1987), and it is sensitive to environmental disturbances (Labbate et al., 2016; Zeng et al., 2019). There are also diversity and compositional differences between the two different bacterial communities (Jiang et al., 2006; Liu et al., 2018; Zeng et al., 2019). Planktonic bacteria can flow to the downstream and benthic zone, where they coalesce with sedimentary bacteria (Mansour et al., 2018; Gao et al., 2021). However, the extent to which these bacterial communities merge and coalesce in aquatic ecosystems is still not fully understood (Mansour et al., 2018; Langenheder and Lindström, 2019). This question includes the merging and coalescence of the same bacterial community in different aquatic environments, and the merging and coalescence of different communities in the same environment.

From an ecological perspective, a community coalescence event is more than just a part of a dispersal process, and it results in interactions between the whole community and its environment (Rillig et al., 2015). Meanwhile, community coalescence is an exchange event among communities (and the surrounding environments); that is, individual communities coalesce with a new entity under mixing of relatively large environments (Rillig et al., 2015). By contrast, bacterial dispersal encompasses the immigration and establishment of individuals (Hanson et al., 2012). In recent years, coalescence of bacterial communities has received increasing attention, including the construction of theoretical frameworks (Rillig et al., 2015; Mansour et al., 2018), verification by microcosmic experiments or mathematical algorithms (Livingston et al., 2013; Rillig and Mansour, 2017), significance in biological evolution (Castledine et al., 2020), and quantitative extent of community coalescence (Zhou and Ning, 2017; Mei and Liu, 2019). However, comprehensive studies elucidate the distribution patterns and ecological significance of bacterial community coalescence in natural habitats are still limited. Consequently, how hydrological connectivity influences bacterial community coalescence in floodplain ecosystems remains an open question.

Coalescence is a community assembly process involving settlement and interactions of species (Castledine et al., 2020). Co-occurrence network analysis is commonly used to explore interactions among species and to ascertain the importance of certain species (Röttjers and Faust, 2018). Co-occurrence networks cannot always illustrate a real biological connection (Freilich et al., 2018; Qiu et al., 2021). Nonetheless, co-occurrence network analysis can visualise the complexity of bacterial communities, and identify which taxa are more important than others for maintaining the network structure (Qiu et al., 2021; Yuan et al., 2021). The network structure of bacterial communities in rivers is influenced by water environmental factors (Peng et al., 2017). Community coalescence is bound to affect the complexity of the bacterial network, the number of keystone species, and the connectivity among species. Thus, network analysis can be used as a tool to determine the possible influence of bacterial community coalescence on interspecies interactions under variable hydrological connectivity.

The present study was conducted in a floodplain ecosystem in the source region of the Yellow River, China. We analyzed the merging and coalescence of planktonic and sedimentary bacterial communities in vertical and lateral directions during different hydrological periods. We hypothesized that coalescence of planktonic and sedimentary bacterial communities occurs in the floodplain during the normal period, and would be enhanced in vertical and lateral directions by increased hydrological connectivity during the high-water period; in this way, hydrological connectivity positively influences the network complexity of bacterial communities and community coalescence, ecologically. To verify the hypothesis, we studied the distribution patterns of different bacterial communities at multiple spatiotemporal scales, quantified the extent of community coalescence, and investigated the influence of community coalescence on bacterial networks. This study was designed to explore the following: (1) Why are there differences in the spatial distribution patterns of bacterial communities between normal and high-water periods? (2) How does hydrological connectivity influence lateral and vertical coalescence of bacterial communities, in addition to the community structure and keystone species? (3) How does community coalescence improve the stability of bacterial communities in the floodplain?

## Materials and methods

### Study area and sampling

**Figure 1:**
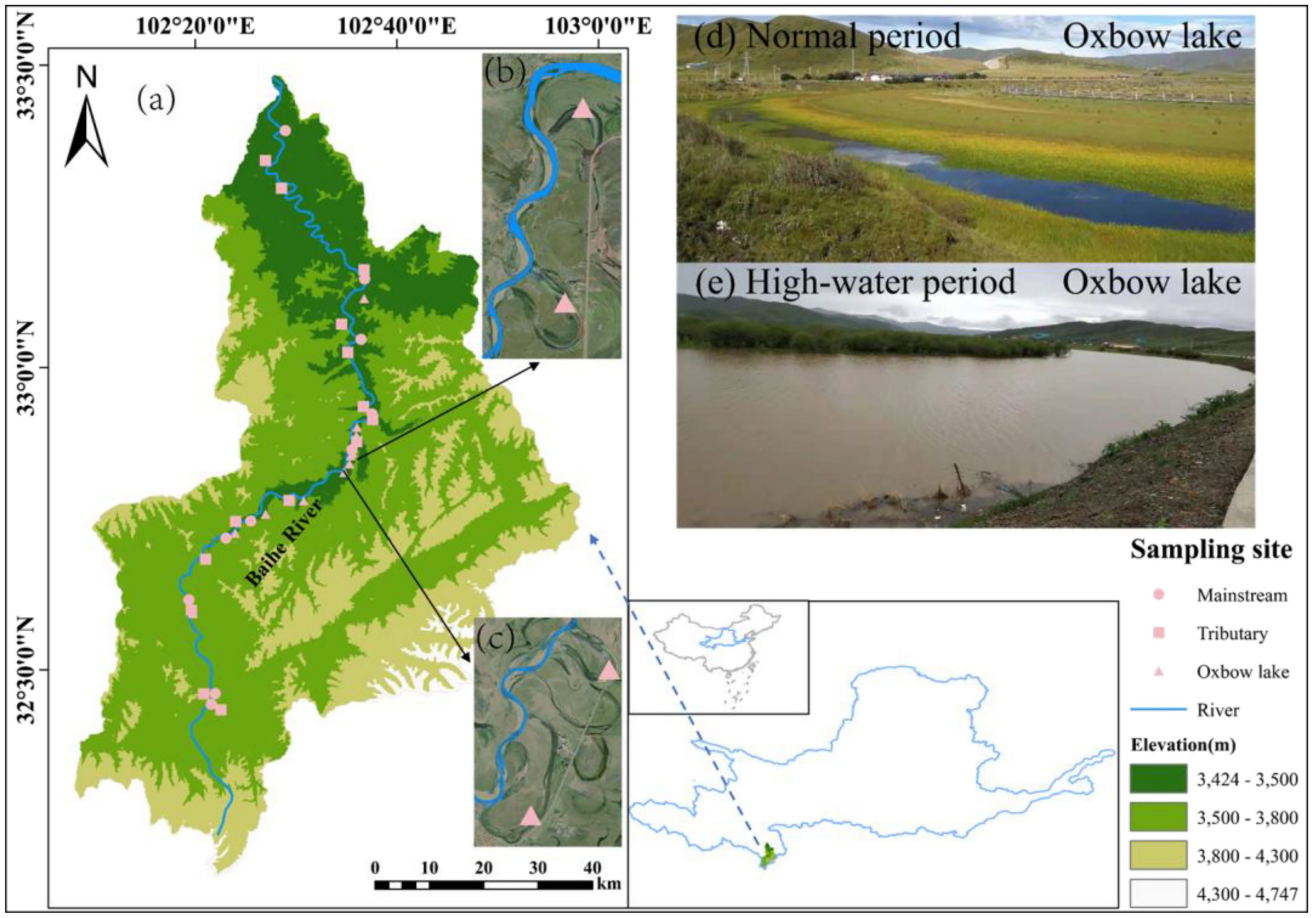
Locations of sampling sites in Baihe River in the source region of the Yellow River, China. **(A)** Baihe River Basin. **(B)** Sampling sites at oxbow lakes 5 and 6. **(C)** Sampling sites at oxbow lakes 7 and 8. **(D)** Oxbow lake in normal period. **(E)** Oxbow lake in high-water period.

The study area (102°00′–103°00′E, 33°00′–33°30′N) is located in the Baihe River Basin in Hongyuan County (Yellow River source region), Aba Tibetan and Qiang Autonomous Prefecture, Sichuan Province, Southwest China. The Baihe River has a large number of tributaries and oxbow lakes, providing a natural observation window for this study. We classified the Baihe River into three types of water bodies (i.e., mainstream, tributaries, and oxbow lakes) based on their connectivity. A total of 36 sampling sites were selected along the Baihe River, with 10 in the mainstream, 14 in the tributaries, and 12 in the oxbow lakes (Figure 1).

Considering the influence of river connectivity on the migration and spread of bacterial communities, paired samples of surface water (0.5 m depth) and surface sediment (0.05 m depth) were collected in September 2019 (normal period: the runoff of the Baihe River is 41.81 ± 23.49 m^3^/s) and June 2020 (high-water period: the runoff is 164.17 ± 136.03 m^3^/s). In each season, sampling was completed within a 5-day period. At each sampling site, water samples (10 L each) were collected using two 5 L sterile polyethylene terephthalate bottles and kept at a low temperature of 0°C–4°C. Meanwhile, three sediment samples were collected near the water sampling site and mixed to form a composite sample, which was sealed in 50 ml sterile polypropylene tubes and kept in liquid nitrogen. All samples were immediately transported to the laboratory, where water samples were filtered through a 0.22 μm polycarbonate membrane (47 mm diameter; Millipore, Billerica, MA, United States). The filtered membranes and sediment samples were stored at-80°C until DNA extraction. A total of 140 samples (72 water and 68 sediment) were collected.

### Environmental information

A total of 23 environmental variables were measured or collected (Supplementary File 2; Supplementary Table S1). Nine of the environmental variables were measured in the field. Specifically, flow velocity (V) was measured using an FP211 direct-reading flow meter (Global Water Instrumentation, Sunnyvale, CA, United States). Water quality parameters, namely water temperature (WT), electrical conductivity (EC), dissolved oxygen (DO), pH, oxidation–reduction potential (ORP), and total dissolved solids (TDS), were measured using a portable multi-parameter analyzer (YSI Corp., Yellow Springs, OH, United States). Turbidity (Tur) was measured with a 2100Q portable turbidity meter (Hach, Loveland, CO, United States), and mud temperature (MT) was measured using a DS600T mud thermometer (EDKORS, Changzhou, Jiangsu Province, China).

Another 14 environmental variables were determined in the laboratory. For water samples, chemical oxygen demand (COD) was determined by fast-digestion spectrophotometry based on the Chinese Environmental Protection Industry Standard for Water Quality (HJ/T 399-2007), and dissolved organic carbon (DOC) was determined by combustion based on the International Standard for Water Quality (ISO 8245-1987). Total phosphorus (TP), total nitrogen (TN), ammonium-nitrogen (NH_4_-N) and nitrate-nitrogen (NO_3_-N) of water samples were determined by spectrophotometry according to standard methods described in “Water and Wastewater Monitoring and Analysis Methods” (Third Edition). Levels of chlorophyll *a* (Chl-*a*) in water samples were determined by spectrophotometry after extraction with 95% ethanol according to “Specifications for Lake Eutrophication Investigation” (Second Edition). Soil total nitrogen (STN), total phosphorus (STP) and organic carbon (SOC) were also determined based on the Chinese Environmental Protection Industry Standards for Soil Quality (HJ 717-2014, HJ 632-2011 and HJ 615-2011, respectively). Sediment particle size was measured using a Mastersizer 2000 Laser Particle Sizer (Malvern Instruments Ltd., Worcestershire, United Kingdom) with a working range of 0.02–2,000 μm and relative error vc < 1%. Median particle size (D_50_) was obtained after drawing a gradation curve. Sediment type with a grain size was classified as clay (particle size <4 μm, 8Φ), silt (4–63 μm, 4–8Φ) and sand (>63 μm, 4Φ; Huang et al., 2010).

### Illumina sequencing and bioinformatics analysis

Genomic DNA was extracted in duplicate using a FastDNA SPIN Kit (MP Biomedicals, Santa Ana, CA, United States) according to the manufacturer’s protocols. Duplicate DNA extracts were pooled for subsequent PCR amplification on a BioRad S1000 (Bio-Rad Laboratory, Hercules, CA, United States), targeting the hypervariable V4 region of the bacterial 16S ribosomal RNA (rRNA) gene. Each DNA sample was amplified using primers 515F (5′-GTGYCAGCMGCCGCGGTAA-3′) and 806R (5′-GGACTACNVGGGTWTCTAAT-3′; Invitrogen, Carlsbad, CA, United States). PCR amplifications contained 25 μl of 2 × Premix Taq (Takara Biotechnology, Dalian, Liaoning Province, China), 1 μl of each primer (10 mM) and 3 μl of sample DNA (20 ng/μl). Thermal cycling included an initial denaturation at 94°C for 5 min, followed by 30 cycles of 30 s at 98°C, 30 s at 52°C, and 30 s at 72°C, and a final extension step of 10 min at 72°C. Triplicate PCR products for each of the 140 samples were purified using an AxyPrep DNA Gel Extraction Kit (Axygen Biosciences, Union City, CA, United States). All libraries were sequenced on an Illumina MiSeq platform (Illumina Inc., San Diego, CA, United States) using a paired-end (2 × 250 bp) approach. The raw 16S rDNA sequence data have been stored in a public National Center for Biotechnology Information (NCBI) database (accession number: PRJNA853875).

Sequences of bacterial 16S rRNA gene amplicons were quality-filtered using QIIME v2.0 (Li et al., 2019) following the official suggestions, and detailed processes can be found elsewhere (Gao et al., 2020). High-quality sequence data were checked and corrected using DADA2 to obtain operational taxonomic units (OTUs) with a sequence similarity of 100% (Gao et al., 2021). Taxonomic annotation of OTUs was assigned using the Naive Bayes classifier trained by the Silva (SSU132) 16S rRNA database (Hoyningen-Huene et al., 2019). In order to prevent sequencing errors in subsequent analyses, all sequences classified as chloroplasts, mitochondria, archaea, or eukaryotes were removed (Mo et al., 2018). Furthermore, to minimize the influence of unequal sequencing efforts, random sampling was conducted on an ESV table to equalize the number of sequences in each sample (*n* = 9,315).

### Data analysis

#### Alpha-and beta-diversity

We calculated alpha-diversity (i.e., OTU richness, Chao1 and Shannon-Wiener indices) of bacterial communities for each sample using vegan version 2.5-7 with R program version 4.1.0 (Chen et al., 2019). One-way analysis of variance (ANOVA) and Student’s *t*-test were used to compare alpha-diversity between groups in SPSS version 25.0 (IBM Corp., Armonk, NY, United States). For beta-diversity, bacterial community composition was visualized using non-metric multidimensional scaling (NMDS) based on Bray-Curtis dissimilarities, and analysis of similarity (ANOSIM) was used to evaluate differences in bacterial communities between groups (Mo et al., 2021). These were implemented using the R program (version 4.1.0) with vegan (version 2.5-7), ggplot2 (version 3.3.5), and RColorBrewer (version 1.1-2) packages.

#### Community coalescence

We used three standard methods to evaluate the immigration and coalescence of bacterial communities. First, R version 4.1.0 was used to calculate the overlap of species (proportion of shared species, or number of reads of common OTUs) between adjacent communities (Gao et al., 2021). Second, the Bayesian classifier SourceTracker was used to predict the contributions of different types of upstream sources to different types of downstream sinks (Knights et al., 2011). Finally, the helperfunctions.r and calcCohesion.r packages were used to quantify the connectivity between communities (Herren and McMahon, 2017).

#### Habitat niche breadth

We calculated the Levins’ niche breadth (*B*) index for bacterial communities using the following formula:


$$B_{j}=\frac{1}{\sum_{i=1}^{N}P_{ij}^{2}}$$

where *B_j_* indicates the habitat niche breadth of OTU *j* in a metacommunity, *N* represents the total number of communities in each metacommunity, and *P_ij_* is the proportion of OTU *j* in community *i* (Wu et al., 2018). A high *B* value represents a wide habitat niche breadth. It is generally believed that at the community level, the wider the niche, the broader the distribution and the larger the number of species, and vice versa (Jiao et al., 2020). The calculation was implemented using the R spaa package (version 0.2.2; Zhang, 2016).

#### Co-occurrence network

The OTU distribution patterns in samples of normal and high-water periods were displayed across the taxonomic tree by directed networks using the *prefuse* layout algorithm in CYTOSCAPE v3.7.1 (Faust and Raes, 2016). We selected prevalent OTUs (present in ≥20% of samples) among samples in the same habitat type as nodes to prevent inconsistent trends caused by transient OTUs (Liu et al., 2018). The network topology of each sample was characterized using the *subgraph* function *via* the R igraph package (Ma et al., 2016), in terms of node number (the number of OTUs), edge number (the number of connections among all nodes), average path length (APL, average shortest path length between any two nodes in the network), and betweenness (the number of times a node acts as a bridge along the shortest path between two other nodes). Higher node number, edge number and APL and lower betweenness represent greater network complexity (Gao et al., 2021; Qiu et al., 2021). Identification of keystone species was based on calculation of within-module connectivity (*Z_i_*) and among-module connectivity (*P_i_*) in the co-occurrence network (Guimerà et al., 2005). Excluding peripherals (*Z_i_* < 2.5, *P_i_* < 0.62), the other three types of nodes (module hubs, connectors and network hubs) were classified as keystone species (Deng et al., 2012; Shi et al., 2016). Visualisation of the co-occurrence network was performed using Gephi version 0.9.2.^1^

## Results

### Comparison of environmental factors between normal and high-water periods

Approximately half of the 23 environmental variables in floodplain showed a significant difference between normal and high-water periods (*p* < 0.05; Supplementary File 2; Supplementary Table S2). In the mainstream, the mean values of EC, TDS, NH_4_-N, TN, and TP were all significantly higher in the normal period than the high-water period, while the opposite was true for ORP and Tur. In tributaries, the mean values of EC, DO, TDS, Si, NH_4_-N and STN were significantly higher in the normal period than the high-water period, in contrast to the trends of ORP, Chl-*a* and Tur. In oxbow lakes, EC, TDS, NH_4_-N, TN and TP displayed similar trends to those in the mainstream, with significantly higher mean values in the normal period than the high-water period. On the contrary, ORP, Tur, STN and STP exhibited higher mean values in the high-water period than the normal period. Overall, the mean values of EC, TDS and NH_4_-N were significantly higher in the normal period than in the high-water period, while only ORP had higher mean values in the high-water period.

### Relative abundances of bacterial communities

A total of 181,778 OTUs were retrieved from the 140 samples by high-throughput sequencing. The rarefaction curves revealed that the bacterial OTUs obtained from the applied sequencing depth were sufficient to represent the bacterial communities in water and sediment samples. In addition, the number of OTUs observed in different times and spaces were highly variable; the number of sedimentary bacterial OTUs was greater than that of planktonic bacterial OTUs, while the number of OTUs in the high-water period was greater than that in the normal period. Specifically, the number of OTUs in different groups were ordered sediment in the high-water period (HS) > water in the high-water period (HW) > sediment in the normal period (NS) > water in the normal period (NW; Supplementary File 1; Supplementary Figure S1).

Regarding planktonic bacteria, irrespective of the season, the number of OTUs in tributaries was the highest, and the number of OTUs in oxbow lakes was the lowest. In the high-water period, the mean number of OTUs in the three different water environments was 2.4 times that in the normal period. Regarding sedimentary bacteria, in both periods, tributaries harboured the largest number of OTUs, with the fewest found in the mainstream. Similar to planktonic bacteria, the number of OTUs in the three different sedimentary environments was higher in the high-water period than in the normal period. However, the magnitude of the increase in OTUs varied in different water body types, by 2.3 times in tributaries, 1.7 times in oxbow lakes, and 1.4 times in the mainstream (Supplementary File 1; Supplementary Figure S2).

**Figure 2:**
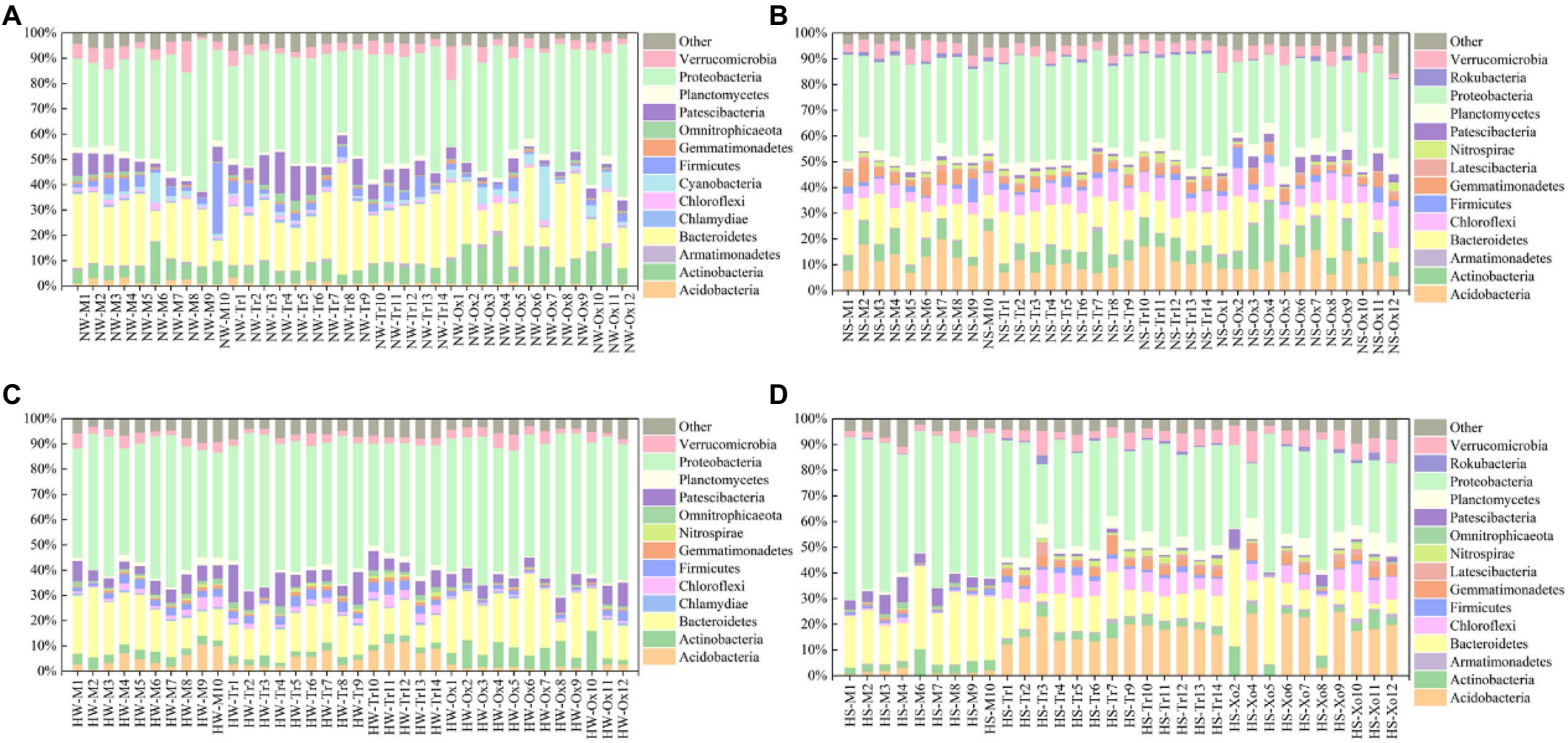
Relative abundances of major bacterial phyla in water and sediment samples of different periods. **(A)** Water in the normal period. **(B)** Sediment in the normal period. **(C)** Water in the high-water period. **(D)** Sediment in the high-water period.

With respect to the relative abundance of major bacterial phyla, Proteobacteria accounted for the largest proportions of planktonic and sedimentary bacterial communities in the two periods, and the proportions in water were slightly larger than those in sediment. Bacteroidetes was the second dominant phylum in all samples. In addition, Chlamydiae and Cyanobacteria only existed in water, while Latescibacteria and Rokubacteria only occurred in sediment. The major bacterial phyla also shifted with season. For example, Armatimonadetes and Cyanobacteria only appeared in the normal period (water), while Nitrospirae only emerged in the high-water period (water and sediment). Compared with the planktonic bacterial community, variations in the sedimentary bacterial community were minimal between the two study periods (Figure 2).

### Diversity of bacterial communities

With the exception of Good’s coverage, the other five alpha-diversity indices of planktonic and sedimentary bacterial communities were all significantly higher in the high-water period than in the normal period (Supplementary File 1; Supplementary Figure S3). For the planktonic bacterial community, the five alpha-diversity indices were highest in tributaries, followed by the mainstream, and lowest in oxbow lakes in both periods. However, the five alpha-diversity indices of the sedimentary bacterial community in the three types of water bodies exhibited distinctively different trends between the two periods. In the normal period, there was little difference among the three sedimentary environments, despite slightly higher bacterial diversity in oxbow lakes and slightly lower bacterial diversity in the mainstream. In the high-water period, tributaries harboured the highest bacterial diversity, while the mainstream showed the lowest bacterial diversity, and there was a significant difference between the mainstream and the other two water body types.

**Figure 3:**
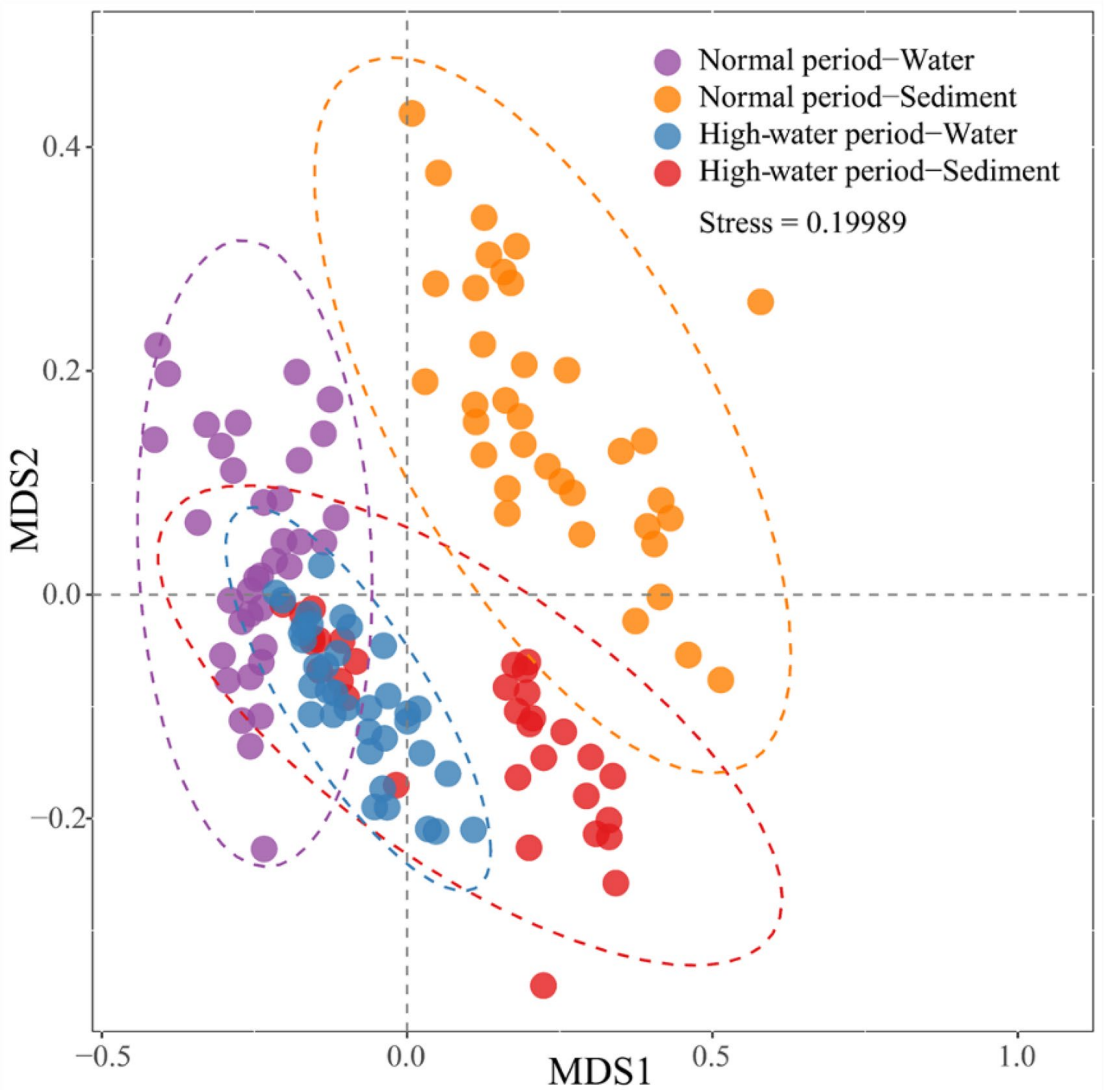
Non-metric multidimensional scaling (NMDS) biplot showing differences in bacterial community composition in water and sediment samples of Baihe River across normal and high-water periods.

The NMDS biplot shows that the bacterial communities of water samples were significantly different from those of the corresponding sediment samples in the normal period, while only partial community differences were observed in the high-water period (Figure 3). The bacterial communities of sediment samples displayed distinct seasonal variations, but the bacterial communities of water samples did not form two separated clusters for the two seasons. The consistency of the results was corroborated by ANOSIM (Supplementary File 1; Supplementary Figure S4). Both the planktonic and sedimentary bacterial communities were significantly different between normal and high-water periods (planktonic, global *r* = 0.203, *p* = 0.001; sedimentary, global *r* = 0.263, *p* = 0.001).

Furthermore, the NMDS and ANOSIM results demonstrated a clear separation of different bacterial communities (NW, NS, HW, and HS) in samples based on water body type (Supplementary File 1; Supplementary Figures S5, S6). For the normal water period, separation of the sedimentary bacterial community was clearer than that of the planktonic bacterial community; however, irrespective of the planktonic or sedimentary bacterial community, oxbow lake samples were markedly different from mainstream and tributary samples (Supplementary File 1; Supplementary Figures S5a,b). For the high-water period, separation of the planktonic bacterial community was clearer than that of the sediment bacterial community, and there were significant differences in the sedimentary bacterial community between the mainstream and tributaries (Supplementary File 1; Supplementary Figures S5c,d). In the high-water period, the planktonic bacterial community in the oxbow lakes and mainstream clustered more closely, while the sedimentary bacterial community of the mainstream and tributaries tended to be separated more clearly, compared with those in the normal period. In addition, the sedimentary bacterial community in different oxbow lake samples showed significant differences in the high-water period (Supplementary File 1; Supplementary Figure S5).

### Coalescence of bacterial communities

Water and sediment from adjacent sampling sites were regarded as sources and sinks for the coalescence of bacterial communities, and default flow directions (from west to east, and from tributaries to mainstream to oxbow lakes) were taken into consideration to obtain more general and meaningful results. The detailed pairs of tributaries–mainstream–oxbow lake samples that met the upstream–downstream requirements are listed in Supplementary File 2; Supplementary Table S3.

**Figure 4:**
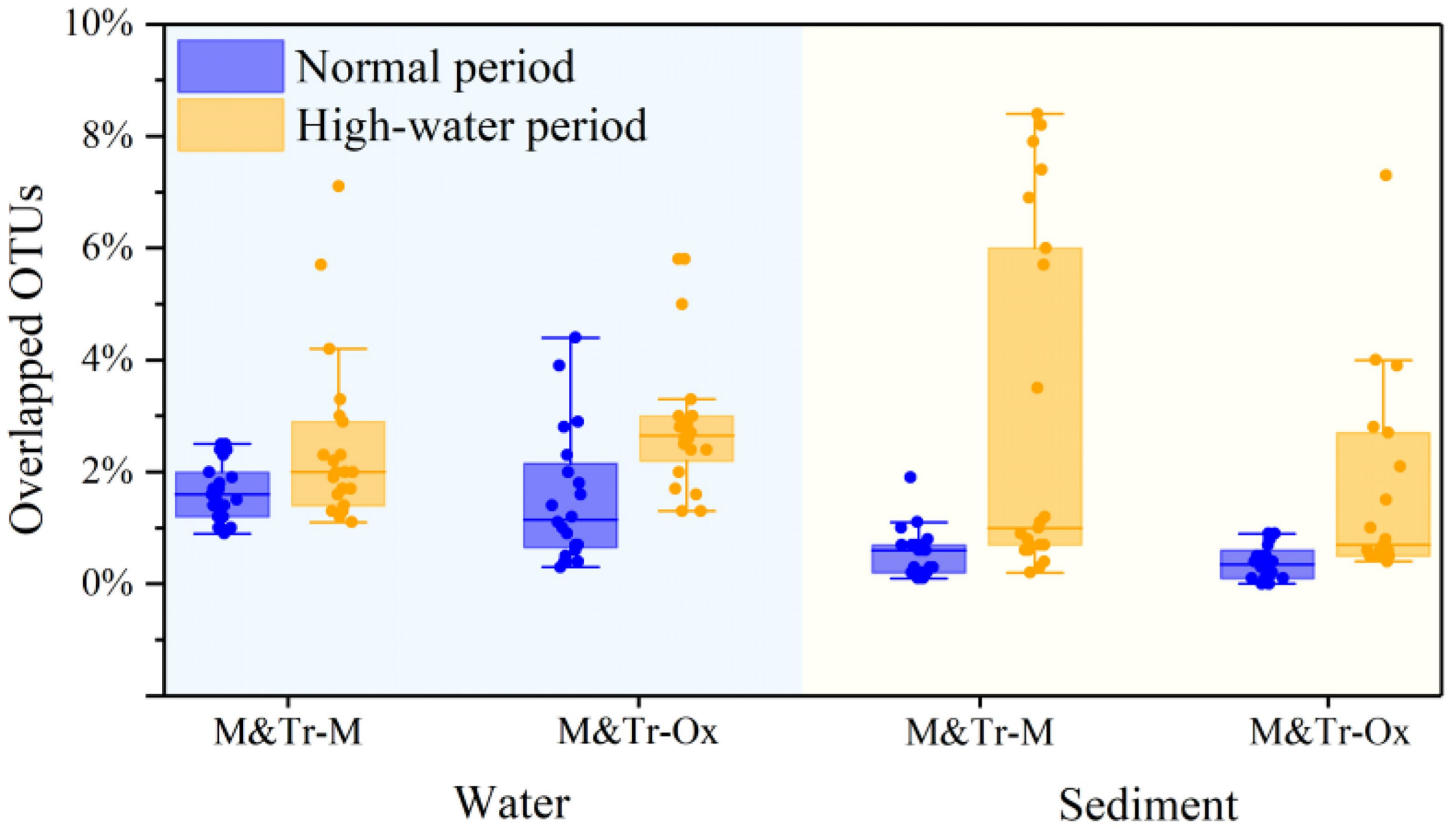
Proportions of overlapping operational taxonomic units (OTUs) between adjacent sampling sites in all OTUs of both sites in different study periods. The proportions of overlapping OTUs were used to quantify community coalescence between upstream and downstream sites. M&Tr-M represents the contribution of the upstream mainstream and tributaries to the downstream mainstream, and M&Tr-Ox represents the contribution of the upstream mainstream and tributaries to the downstream oxbow lakes. Data are means ± standard deviation.

Based on this hypothesis, the relative abundance of overlapping (shared) OTUs was calculated for each bacterial community and its neighbored upstream communities (Figure 4). Following merging of upstream–downstream bacterial communities in pairs, OTUs in water were more preserved than those in sediment, irrespective of the season. This indicates greater coalescence of planktonic bacteria than for sedimentary bacteria across different periods. Compared with the normal period, preservation of OTUs in both water and sediment was higher in the high-water period. Accordingly, there was increased connectivity between the tributaries, mainstream, and oxbow lakes in the high-water period, which promoted the integration of bacterial communities.

**Figure 5:**
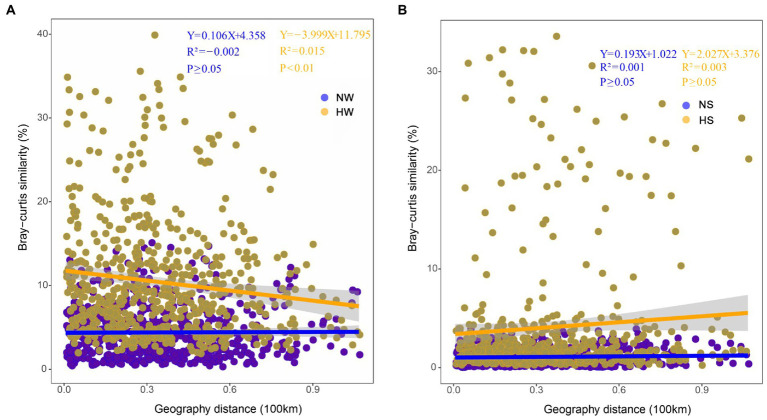
Distance-decay patterns based on the Bray–Curtis similarity of bacterial community composition and cumulative dendritic distance in different periods. **(A)** Comparison of planktonic bacterial community between the two periods. NW, water in the normal period; HW, water in the high-water period. **(B)** Comparison of sedimentary bacterial community between the two periods. NS, sediment in the normal period; HS, sediment in the high-water period.

The same trends were found based on correlation analysis between Bray–Curtis similarity matrices of bacterial communities and cumulative dendritic distances. The planktonic bacterial community displayed a distance attenuation pattern during the high-water period (*p* < 0.01; Figure 5). In the vertical direction, the coalescence of bacterial communities in water and sediment also showed temporal and spatial differences; with increasing water level, bacterial communities in the mainstream water and sediment merged most strongly (normal period, 0.23% ± 0.07%; high-water period, 2.94% ± 0.35%; Supplementary File 1; Supplementary Figure S7).

**Figure 6:**
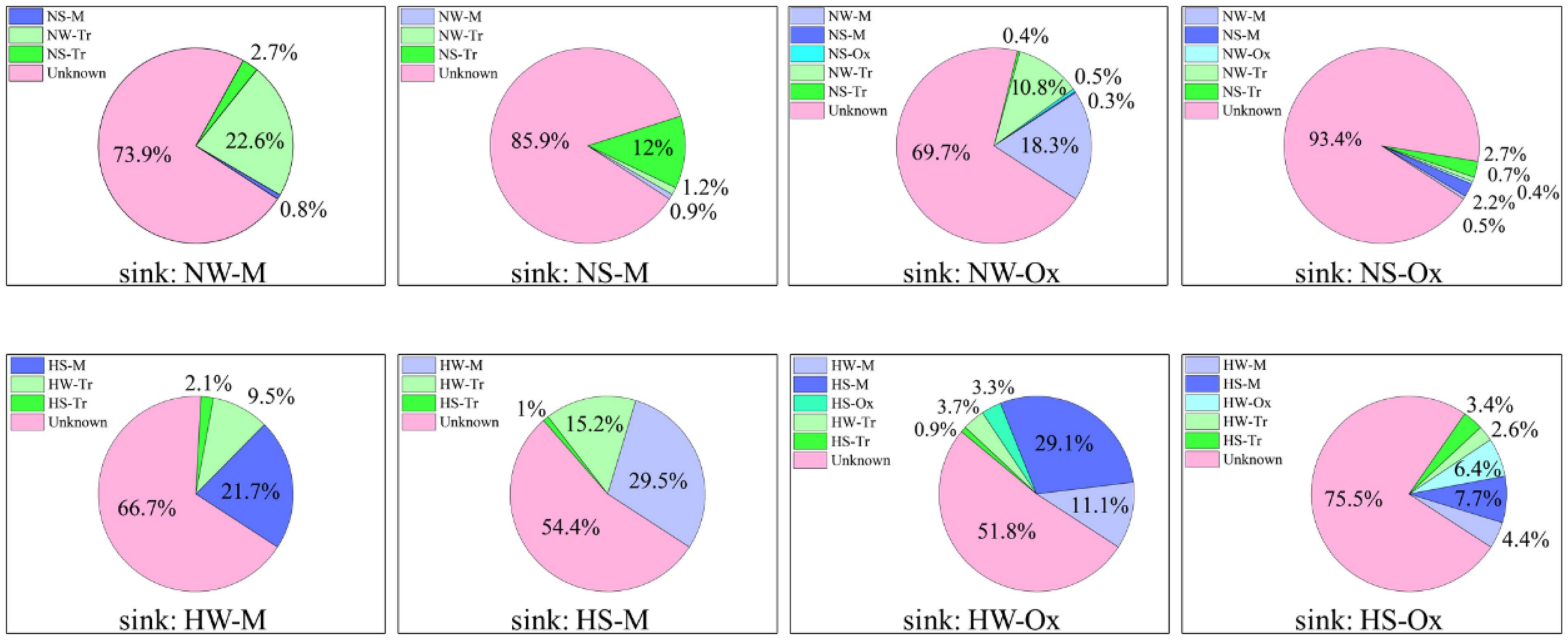
SourceTracker estimates of the contributions of source communities to sink communities of planktonic and sedimentary bacteria in the mainstream and oxbow lakes of Baihe River across different periods. NW, water in the normal period; NS, sediment in the normal period; HW, water in the high-water period; HS, sediment in the high-water period; M, mainstream; Tr, tributaries; Ox, oxbow lakes.

The coalescence patterns of bacterial communities were corroborated by SourceTracker estimates (Figure 6). In the normal period, when the sink was set as the mainstream water or sediment, the planktonic and sedimentary bacterial communities in tributaries made larger contributions, respectively. Under the influence of connectivity, the bacterial communities in both the mainstream and tributaries were major contributors when the sink was set as oxbow lake water or sediment. When compared between water and sediment, there was greater coalescence between planktonic bacterial communities than between sedimentary bacterial communities. In the high-water period, the source of the bacterial community changed, and the contribution of the bacterial community in the mainstream increased for both planktonic and sedimentary bacteria.

**Figure 7:**
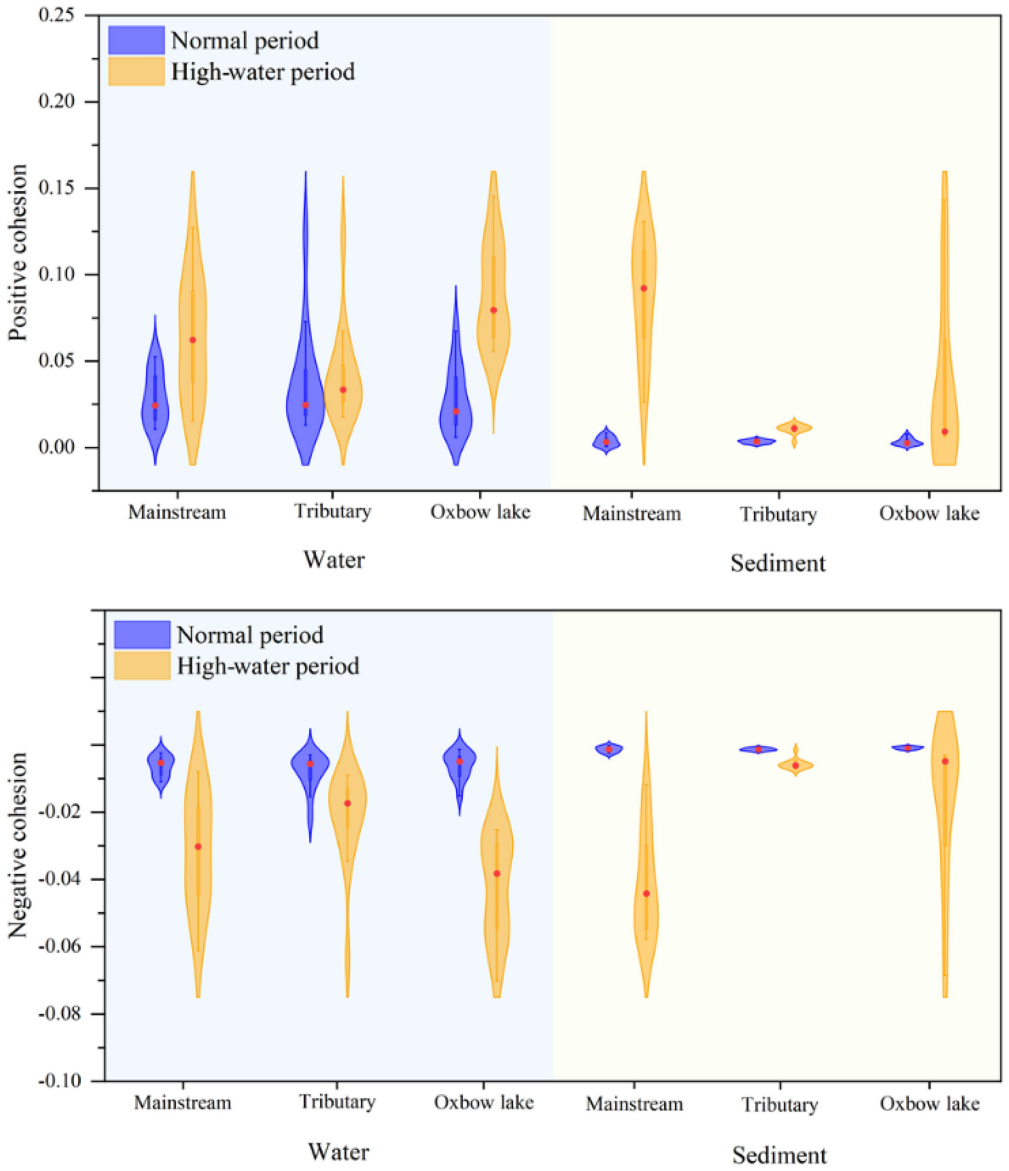
Cohesion metric of bacterial communities in water and sediment samples of the mainstream, tributaries, and oxbow lakes between two different periods.

Next, we calculated the cohesion of bacterial communities across time and space. The absolute values of both positive and negative cohesions of bacterial communities were higher in the high-water period than in the normal period, irrespective of water body type (Figure 7). The results of positive cohesion were consistent with the changes in the mean habitat niche breadth of bacterial communities (Supplementary File 1; Supplementary Figure S8). In the high-water period, the cohesion of both planktonic and sedimentary bacterial communities showed spatial differences. For example, the cohesion of the planktonic bacterial community in oxbow lakes reached the highest level, and the cohesion of the sedimentary bacterial community in the mainstream was much higher than that in the tributaries and oxbow lakes after flooding.

### Co-occurrence patterns and keystone species of bacterial communities

**Figure 8:**
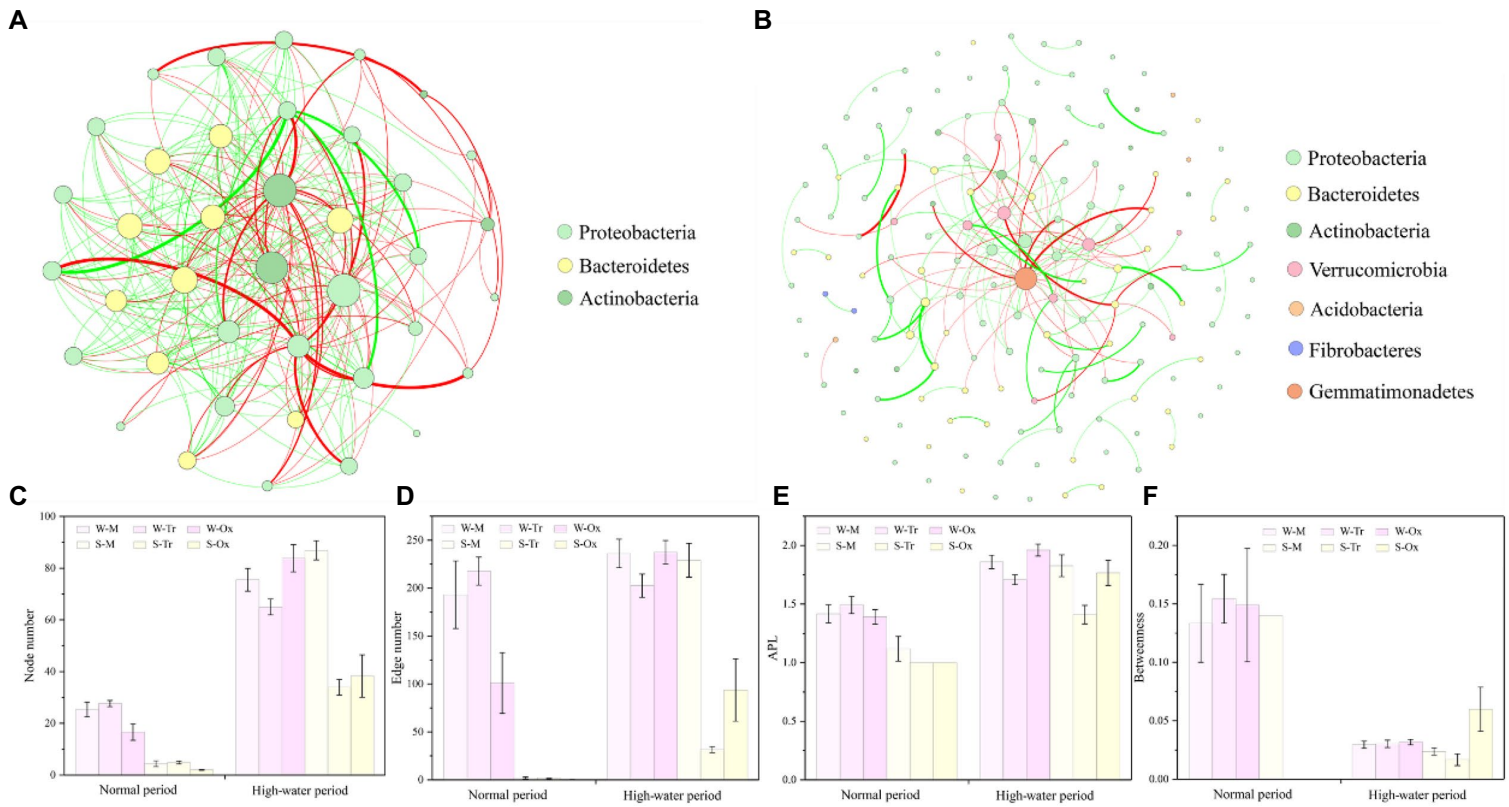
Co-occurrence networks of bacterial communities and topological features of sub-networks. Bacterial networks in the **(A)** normal and **(B)** high-water periods were constructed at the phylum level. The network topology was characterized using **(C)** node number, **(D)** edge number, **(E)** average path length (APL) and **(F)** betweenness. W-M, water-mainstream; W-Tr, water-tributaries; W-Ox, water-oxbow lakes; S-M, sediment-mainstream; S-Tr, sediment-tributaries; and S-Ox, sediment-oxbow lakes.

The networks of bacterial communities constructed for the two different periods demonstrated distinct co-occurrence patterns (Figures 8A,B). In both periods, the betweenness of bacterial networks in water was lower than that in sediment, while their node number, edge number, and APL were all higher than those in sediment (Figures 8C–F). In the normal period, planktonic bacteria dominated the network, which played greater roles in the tributaries than in the mainstream and oxbow lakes. Compared with the normal period, the complexity of the bacterial network increased in the high-water period, and the role of sedimentary bacteria was enhanced, especially in the mainstream.

Network analysis identified 39 OTUs and 367 edges in the bacterial network of the normal period, compared with 159 OTUs and 400 edges in the bacterial network of the high-water period. In both periods, the top three phyla with the largest proportions were Proteobacteria (normal period 64.1%, high-water period 58.49%), Bacteroidetes (25.64, 25.79%) and Actinobacteria (10.26, 6.29%; Figures 8A,B). In the two networks, 39 keystone OTUs were identified for the normal period compared with 112 for the high-water period (Supplementary File 2; Supplementary Table S4). After screening, Proteobacteria, Bacteroidetes and Actinobacteria were the three most abundant phyla, regardless of the water body type, habitat environment, and season. In the normal period, the abundance of keystone OTUs in sediment was extremely low, while the keystone OTUs in water were almost twice as abundant in tributaries than in the mainstream and oxbow lakes. The abundance of keystone OTUs increased dramatically in the high-water period compared with the normal period, but the magnitude of the increase was variable across different water bodies and environments due to distinctive connectivity. The most prominent increases were observed in water of oxbow lakes and sediment of the mainstream (Supplementary File 1; Supplementary Figure S9).

## Discussion

Hydrological connectivity is defined as the amount of water-mediated transfer of matter, energy and organisms within or between elements of the hydrologic cycle (Michaelides and Chappell, 2010). Although hydrological connectivity is one of the main non-biological factors driving ecological processes and organism distribution, its influence on bacterial community coalescence in floodplain ecosystems is largely unknown. In this work, we found that the extent of enrichment and the composition of planktonic and sedimentary bacterial communities vary in different water bodies of a floodplain ecosystem over normal and high-water periods, with hydrological connectivity being the crucial factor driving bacterial community coalescence.

### Bacterial communities display spatiotemporal patterns in the floodplain ecosystem

As expected, NMDS analysis revealed a separate clustering of planktonic and sedimentary bacterial communities in the floodplain of Baihe River in the normal period, with community intersection in the high-water period (Figure 3). This result is contradictory to the findings of Liu et al. (2018) showing that planktonic and sediment bacterial communities did not intersect in the Yangtze River due to seasonal changes. The differences in bacterial communities may be attributable to variations in environmental and hydrographic conditions. The Baihe River mainstream in the present study area was shallow (mean depth 30 ± 15 cm in the high-water period); however, compared with that of the normal period, the mainstream flow velocity drastically increased during the high-water period (Supplementary File 2; Supplementary Table S2), thus contributing to sediment disturbance and hence the coalescence between planktonic and sedimentary bacterial communities. In addition, the number of bacterial OTUs, relative abundances of major taxa (phylum level; Figure 2), and alpha-diversity of bacterial communities were all higher in the high-water period than the normal period (Supplementary File 1; Supplementary Figures S2, S3). These seasonal patterns could be explained by several reasons. First, the bacterial communities may have experienced seasonal succession. Second, in the high-water period, sediment disturbance and the coalescence between planktonic and sedimentary bacterial communities could lead to an increased number of bacterial OTUs and improved diversity. Third, rainfall events might wash out bacteria from the surroundings, increasing species richness and shifting the bacterial communities in the rainy season (Chen et al., 2019).

Among the three typical water bodies (i.e., mainstream, tributaries, and oxbow lakes) of Baihe River, the distribution, number of OTUs, and diversity of planktonic and sedimentary bacterial communities all changed in the study periods (Supplementary File 1; Supplementary Figures S2, S3, S5). The community differences among these water bodies may be partially caused by environmental changes across seasons (Chen et al., 2019; Luo et al., 2019; Gao et al., 2020), as distinctive differences in some environmental variables (e.g., flow velocity, pH, nutrients) were detected (Supplementary File 2; Supplementary Table S2). Another possible reason is the potential influence of hydrological connectivity, because the mainstream, tributaries and oxbow lakes would be connected with each other, and the exchange of matter, energy and species in water would be higher than that of sediment during the high-water period. Furthermore, biological differences (e.g., planktonic and sedimentary bacteria) and interactions between external and internal factors (e.g., physicochemical factors and bacterial species) can enhance bacterial community dynamics (Sommer et al., 2012). Overall, complex interactions among aquatic environments, biological conditions, and spatial factors result in the distinctive patterns of bacterial community diversity and composition in the floodplain ecosystem.

### Hydrological connectivity facilitates bacterial immigration and community coalescence

Similar to previous findings for large rivers (e.g., Liu et al., 2018), the immigration ability of the planktonic bacterial community was higher than that of the sedimentary bacterial community in the floodplain of Baihe River. This phenomenon depends not only on the living habits of bacterial species themselves, but also on the influence of surrounding environments (Liu et al., 2018; Gao et al., 2020). During the high-water period, the connections between the mainstream, tributaries and oxbow lakes would be enhanced with the rising water level. Consequently, the Bray–Curtis similarity of the planktonic bacterial community increased (Figure 5), and planktonic bacteria aggregated in oxbow lakes with increasing flow (Figures 4, 6). However, immigration and coalescence of the sedimentary bacterial community showed different patterns compared with to those of the planktonic bacterial community, consistent with results reported for the Yangtze River (Gao et al., 2021). Accordingly, increased hydrological connectivity in the high-water period can promote the immigration and coalescence of the planktonic rather than the sedimentary bacterial community in the lateral direction of the floodplain.

In the vertical direction, there were spatiotemporal variations in the proportions of overlapping OTUs between planktonic and sedimentary bacterial communities (Supplementary File 1; Supplementary Figure S7). Irrespective of the water body type (i.e., mainstream, tributaries or oxbow lakes), both the planktonic and sedimentary bacterial communities exhibited minimal coalescence in the normal period, in agreement with results for the Yangtze River and other places (e.g., Liu et al., 2018). However, upon the arrival of the high-water period, there was increased coalescence of both planktonic and sedimentary bacterial communities, especially in the mainstream, compared with that of the normal period (Supplementary File 1; Supplementary Figure S7). In addition, the results of microbial source tracing indicated the coalescence of the planktonic bacterial community with the sedimentary bacterial community in the mainstream during the high-water period (Figure 6). These results are mainly attributable to the influence of flood tides, increased mainstream velocity, and suspension of clay and silt in sediment during the high-water period (Padding and Louis, 2004; Zeng et al., 2015).

Furthermore, in the high-water period, suspended sedimentary bacteria would immigrate to the oxbow lakes with flow laterally, while the contribution of tributary bacterial communities to mainstream and oxbow lake bacterial communities showed a downward trend (Figure 6). In summary, hydrological connectivity can facilitate the coalescence of planktonic and sedimentary bacterial communities in the mainstream vertically, and increase the probability of sedimentary bacterial community immigrating from the mainstream to oxbow lakes. As a result of community immigration and coalescence, a more alike community and more homogeneous environment would be formed in the mainstream, tributaries, and oxbow lakes, leading to the convergence of environmental conditions in the floodplain ecosystem.

### Hydrological connectivity influences bacterial network complexity and keystone species

Co-occurrence network analysis can be used to explore interactions between microbial species (Röttjers and Faust, 2018). Compared with that of the normal period, the network of planktonic and sedimentary bacterial communities in the high-water period was more complex mainly because of the increased complexity of sub-networks (Figure 8). A plausible mechanism is that source limitation played a reduced role in the high-water period (e.g., increased availability of nutrients in water and sediment); consequently, the diversity of bacterial species and the complexity of the bacterial network increased (Barberán et al., 2011; Hu et al., 2018; Banerjee et al., 2019). This mechanism is supported by previous observations in rivers showing that microbial network complexity is positively correlated with sediment organic matter (Fagervold et al., 2014) and negatively correlated with water pollution level (Wu et al., 2019). Some researchers have reported that during high-water periods, matter, energy and organic substances within the hydrological cycle can readily transfer between each other, increasing the utilization efficiency of resources by living creatures (Tang et al., 2020; Xie et al., 2020). Therefore, our hypothesis proves that the complexity of the bacterial network increases in the floodplain as a result of increased hydrological connectivity.

Based on the connectivity within and among modules, we identified highly connected bacteria, known as keystone species, in the sub-networks. Keystone species play a key role in the overall structure of the microbiota, and they can be used as indicators of environmental changes (Berry and Widder, 2014; Gao et al., 2021). Therefore, we also investigated the relationships between keystone species and hydrological connectivity in the floodplain ecosystem. Across different habitat environments and seasons, the top three most abundant keystone species both in planktonic and sedimentary bacterial communities were always identified as Proteobacteria, Bacteroidetes and Actinobacteria (Supplementary File 1; Supplementary Figure S9). This result suggests that the keystone bacterial species did not shift with hydrological connectivity in the study area. Previous studies also showed that external factors, including the environment (Wu et al., 2019; Tang et al., 2020) and altitude (Lee et al., 2012) had a profound influence on riverine bacterial species. With respect to different water body types, the keystone species abundances of both planktonic and sedimentary bacterial communities increased in the high-water period compared with those of the normal period (Supplementary File 1; Supplementary Figure S9). The difference is related not only to the seasonal succession of bacterial communities themselves, but also their immigration and coalescence driven by hydrological connectivity. Moreover, a stronger coalescence of keystone species in different habitats (water and sediment) could be supported by the drastic increase in the keystone species abundance of mainstream sediment (Gao et al., 2021). Oxbow lakes, located at the end of the mainstream and tributaries in the lateral flow during the high-water period, are the sites of pooling of keystone species.

We found that some other keystone species in the bacterial sub-networks had higher abundances during the high-water period than the normal period. These keystone species were classified as Verrucomicrobia, Acidobacteria, Fibrobacteres and Gemmatimonadetes, all found in terrestrial habitats including farmland, forest and woodland (Ludwig et al., 2015). Indeed, these land use types were observed around the sampling sites, suggesting the possibility of bacterial community coalescence between aquatic and terrestrial habitats (Mansour et al., 2018), particularly during flood events.

### Ecological implications of bacterial community coalescence promoted by hydrological connectivity

Positive cohesion indicates the extent of cooperative behavior between microbial communities in samples, while negative cohesion reflects competitive behavior among community members (Herren and McMahon, 2017). The results of the present study showed that during the normal period, positive cohesion of the planktonic bacterial community was higher than that of the sedimentary bacterial community in different water body types (Figure 7), consistent with the findings reported for China’s Three Gorges Reservoir (Gao et al., 2021). During the high-water period, there was a higher positive cohesion for bacterial communities in the mainstream and oxbow lakes compared with tributaries (Figure 7), because the mainstream and oxbow lakes were the areas where bacterial communities coalesced. This demonstrates that in the mainstream and oxbow lakes, the coalescence of bacterial communities led to an increase in their positive cohesion, while community complexity and stability increased simultaneously.

The results of bacterial community connectivity quantified using cohesion were corroborated through calculations of niche breadth. During the high-water period, we observed the largest increase in niche breadth for the sedimentary bacterial community in the mainstream and the planktonic bacterial community in oxbow lakes, compared with those of the normal water period (Supplementary File 1; Supplementary Figure S8). Coalescence of bacterial communities could play a positive role in improving competitiveness, expanding the distribution area, and increasing biomass. Furthermore, stronger community coalescence could result in more similar bacterial communities and associated environments (Rillig et al., 2015), with minor changes in community structure and species turnover (Hengeveld, 2002). Our study demonstrates that hydrological connectivity in the floodplain ecosystem facilitates the coalescence of planktonic and sedimentary bacterial communities, and thereby drives homogenous selection, reaching a balance in competition, dispersal, coalescence and selection. Consequently, coalescence of bacterial communities could increase community complexity and stability, thereby enhancing their competition and dispersal capacity.

## Conclusion

We analyzed the spatiotemporal patterns and coalescence processes of planktonic and sedimentary bacterial communities in a floodplain ecosystem of the Yellow River source region. The results highlighted the importance of hydrological connectivity in bacterial community coalescence in the mainstream, tributaries and oxbow lakes. Hydrological connectivity promoted the lateral immigration and coalescence of planktonic bacterial community, and increased its vertical coalescence with sedimentary bacterial community, with plenty of keystone species enriched in the oxbow lakes after coalescence. Furthermore, the coalescence of bacterial communities enhanced the community complexity and stability, thereby improving their competitiveness and dispersal capacity. The findings shed light on the ecological significance of bacterial community coalescence driven by hydrological connectivity in the floodplain ecosystem.

Despite being successful in demonstrating the role of hydrological connectivity in promoting bacterial community coalescence, we did not further explore its influence based on the strength of hydrological connectivity in the oxbow lakes and mainstream. In addition, the shifts in bacterial functions as a result of community coalescence were not taken into consideration. To gain a full understanding of the ecological role of hydrological connectivity in bacterial community coalescence and after coalescence, future studies should quantify the strength of hydrological connectivity in different water body types, and determine how bacterial community coalescence influences bacterial functions in the floodplain ecosystem.

## Data availability statement

The original contributions presented in the study are included in the article/Supplementary material, further inquiries can be directed to the corresponding author.

## Author contributions

BP: methodology, writing–original draft preparation, and formal analysis. QC: visualization and investigation. XL: conceptualization, validation, data curation, and funding acquisition. HS: supervision. XZ: software. ZH: writing–reviewing and editing. All authors contributed to the article and approved the submitted version.

## Funding

This work was supported by the National Natural Science Foundation of China (51939009; 52121006; 51622901; and 92047303).

## Conflict of interest

The authors declare that the research was conducted in the absence of any commercial or financial relationships that could be construed as a potential conflict of interest.

## Publisher’s note

All claims expressed in this article are solely those of the authors and do not necessarily represent those of their affiliated organizations, or those of the publisher, the editors and the reviewers. Any product that may be evaluated in this article, or claim that may be made by its manufacturer, is not guaranteed or endorsed by the publisher.

## Footnotes

^1^ https://gephi.org/
