# Supplementary material for: Hydrological connectivity promotes coalescence of bacterial communities in a floodplain
Source: Front Microbiol. 2022 Sep 21;13:971437. doi: 10.3389/fmicb.2022.971437 (PMC9532515; doi:10.3389/fmicb.2022.971437)
Supplement: Supplementary file 2 [file Data_Sheet_1.docx]

**Supplementary material**

**Hydrological connectivity promotes** **coalescence of** **bacterial communities in a floodplain**

Baozhu Pan^a^, Xinyuan Liu^a^, Qiuwen Chen^b*^, He Sun^c^, Xiaohui Zhao^a^, Zhenyu Huang^a^

^a^ *State Key Laboratory of Eco-hydraulic in Northwest Arid Region of China, Xi'an University of Technology, Xi'an, 710048, Shaanxi, China.*

^b^ *State Key Laboratory of Hydrology-Water Resources and Hydraulic Engineering, Nanjing Hydraulic Research Institute, Nanjing, 210029, China.*

^c^ *State Key Laboratory of Crop Stress Biology in Arid Areas, Shaanxi Key Laboratory of Agricultural and Environmental Microbiology, College of Life Sciences, Northwest A&F University, Yangling, 712100, Shaanxi, China*

**Keywords:** Community coalescence, Co-occurrence network, Hydrologic connectivity, River floodplain, Planktonic bacteria, Sedimentary bacteria

*** Correspondence:**

E-mail: qwchen@nhri.cn

**This additional information contains:**

- - 7 Pages
  - 9 Figure

**Table of Contents**

**Fig. S1** Rarefaction curves of similarity-based operational taxonomic units (OTUs) at the 97% sequence similarity level for bacterial communities in the floodplain ecosystem of Baihe River.

**Fig. S2** Venn diagrams showing the number of operational taxonomic units (OTUs) observed in the mainstream, tributaries, and oxbow lakes at different times and spaces.

**Fig. S3** Variations in alpha-diversity of bacterial communities across different water bodies, habitat environments, and seasons.

**Fig. S4** Rankings for dissimilarities within and between groups for planktonic (a) and sedimentary (b) bacterial communities estimated by analysis of similarity (ANOSIM).

**Fig. S5** Non-metric multidimensional scaling (NMDS) biplots showing the variations in bacterial community composition across three habitat environments in Baihe River.

**Fig. S6** Rankings for dissimilarities within and between groups for planktonic and sedimentary bacterial communities estimated by analysis of similarity (ANOSIM).

**Fig. S7** Proportions of overlapping operational taxonomic units (OTUs) between water and sediment samples at each sampling site relative to total OTUs for both categories.

**Fig. S8** Comparison of mean habitat niche breadth of planktonic and sedimentary bacterial communities in the mainstream, tributaries and oxbow lakes in different periods.

**Fig. S9** Abundances of keystone taxa in bacterial communities at the phylum level for six sub-networks.

**
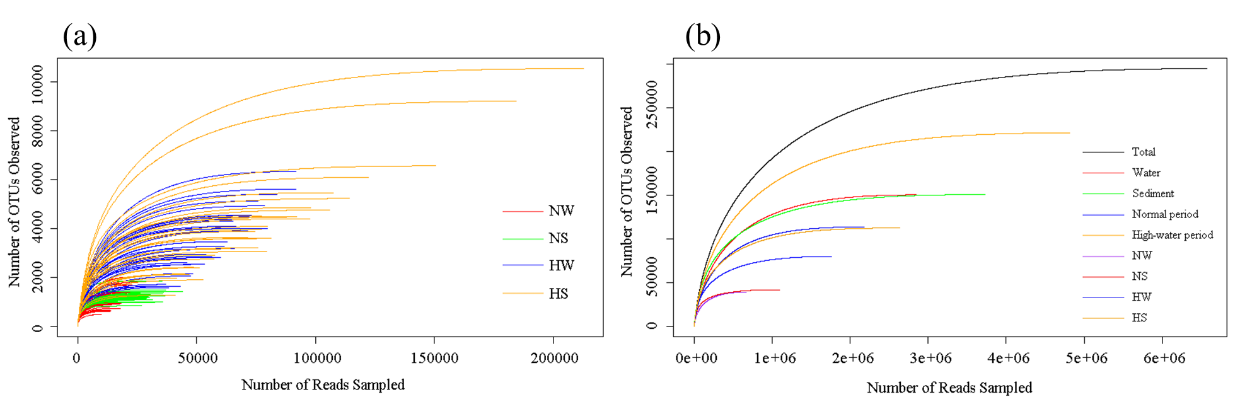
**

**Fig. S1** Rarefaction curves of similarity-based operational taxonomic units (OTUs) at the 97% sequence similarity level for bacterial communities in the floodplain ecosystem of Baihe River. (a) Individual samples. (b) Combined samples.


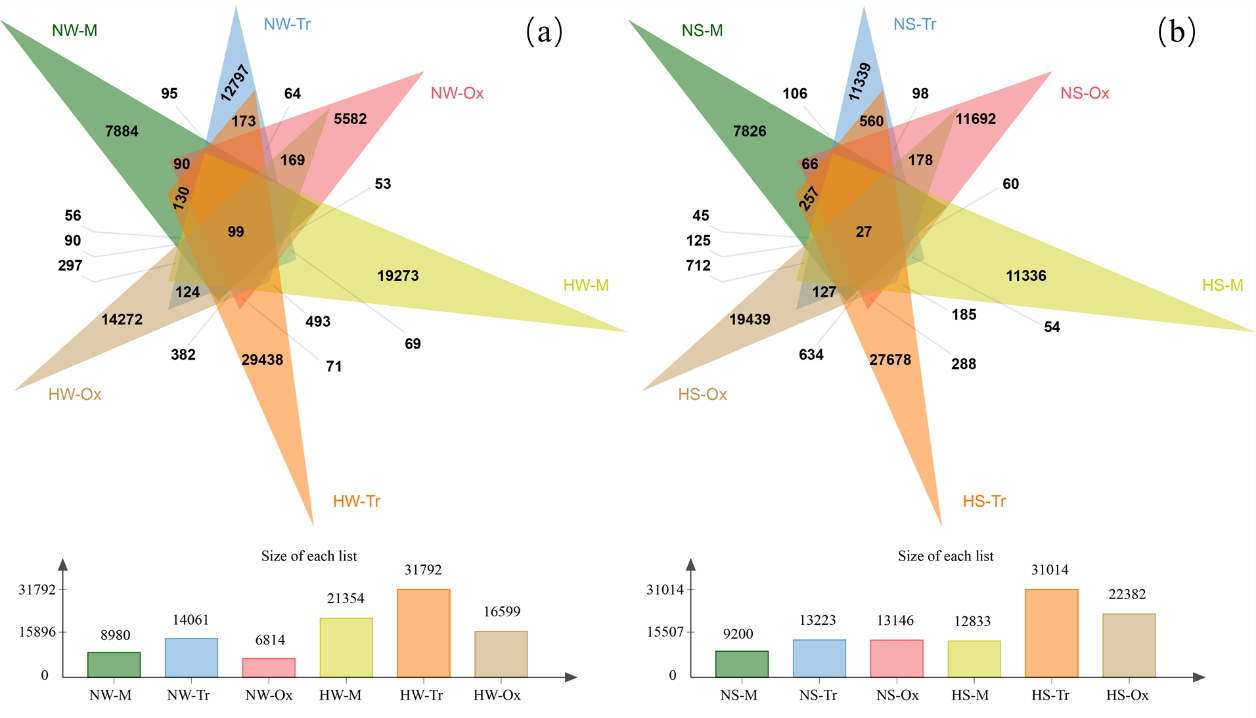


**Fig. S2** Venn diagrams showing the number of operational taxonomic units (OTUs) observed in the mainstream, tributaries, and oxbow lakes at different times and spaces. (a) Three water environments in different periods. (b) Three sediment environments in different periods. NW, water in the normal period; HW, water in the high-water period; NS, sediment in the normal period; HS, sediment in the high-water period; M, mainstream; Tr, tributaries; Ox, oxbow lakes.


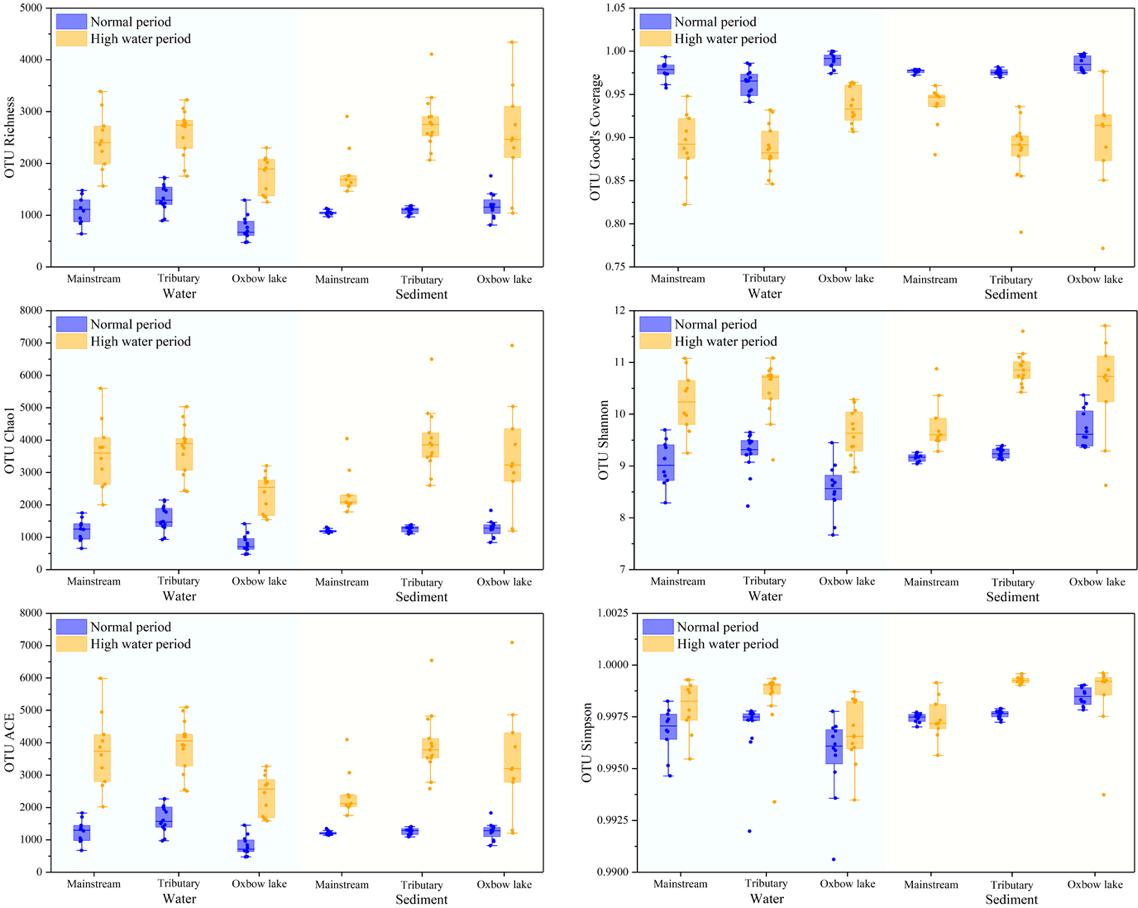


**Fig. S3** Variations in alpha-diversity of bacterial communities across different water bodies, habitat environments, and seasons.


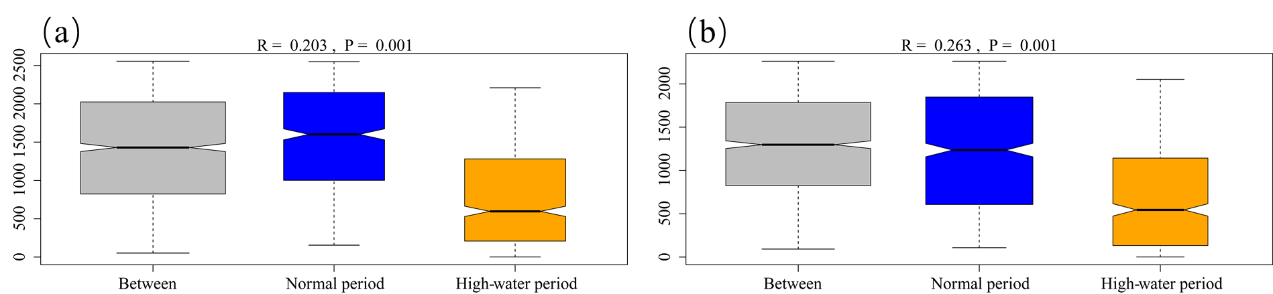


**Fig. S4** Rankings for dissimilarities within and between groups for planktonic (a) and sedimentary (b) bacterial communities estimated by analysis of similarity (ANOSIM). Samples are grouped by season.


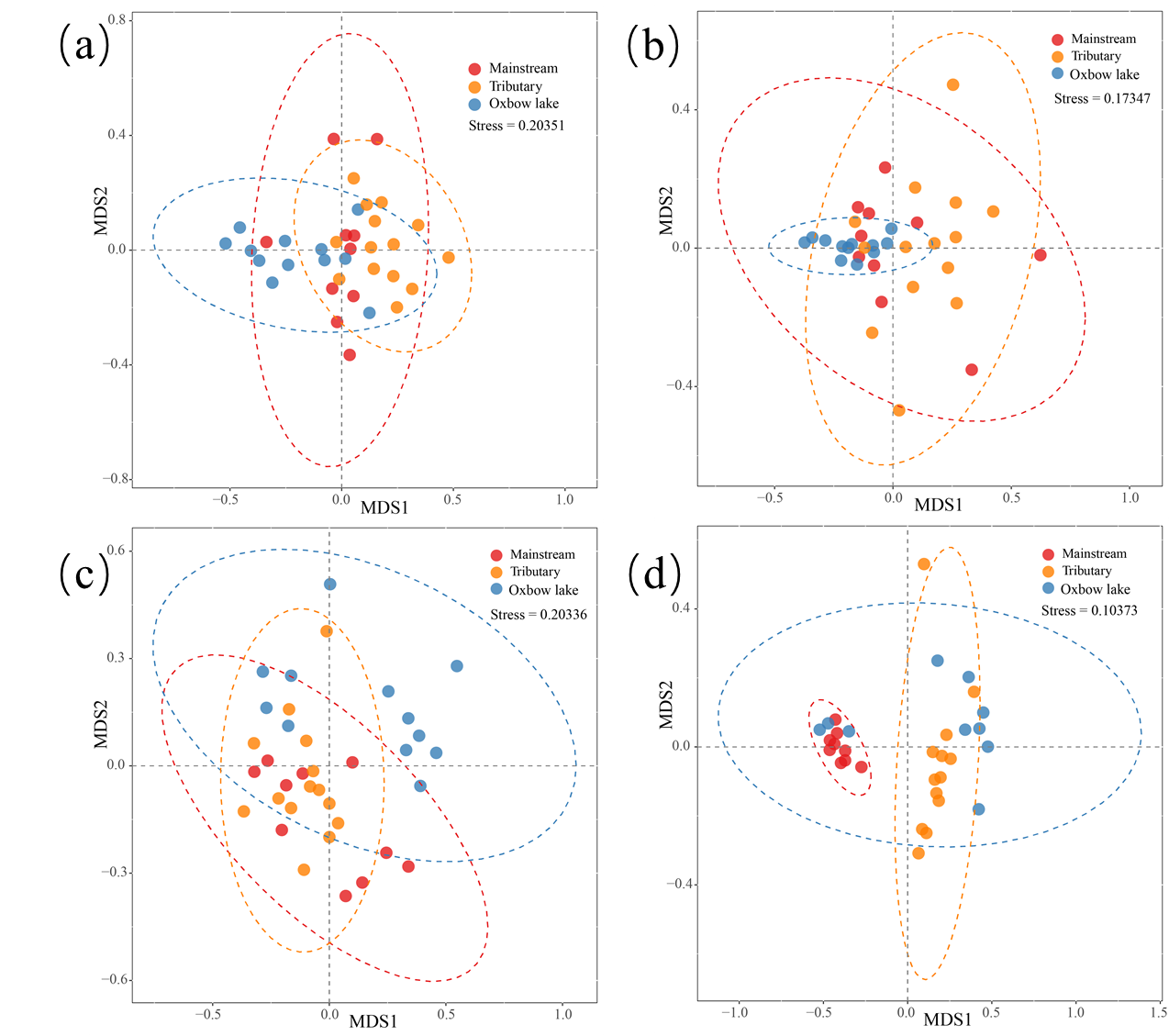


**Fig. S5** Non-metric multidimensional scaling (NMDS) biplots showing the variations in bacterial community composition across three habitat environments in Baihe River. (a) Water in the normal period. (b) Sediment in the normal period. (c) Water in the high-water period. (d) Sediment in the high-water period.


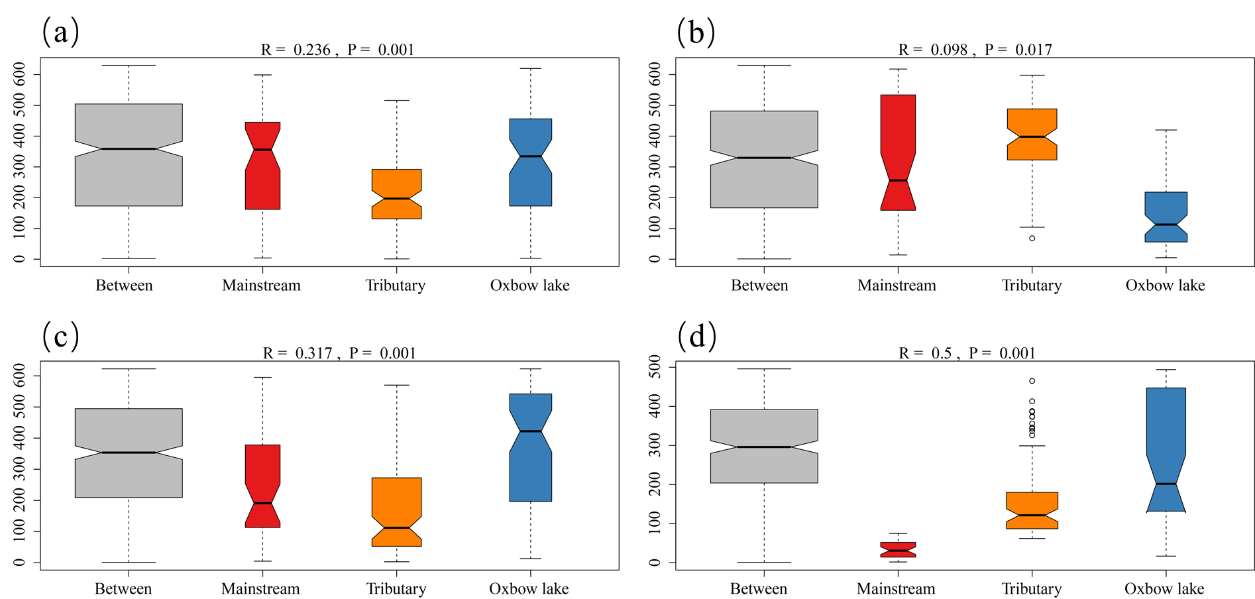


**Fig. S6** Rankings for dissimilarities within and between groups for planktonic and sedimentary bacterial communities estimated by analysis of similarity (ANOSIM). (a) Water in the normal period. (b) Sediment in the normal period. (c) Water in the high-water period. (d) Sediment in the high-water period. Samples are grouped by season and habitat environment.


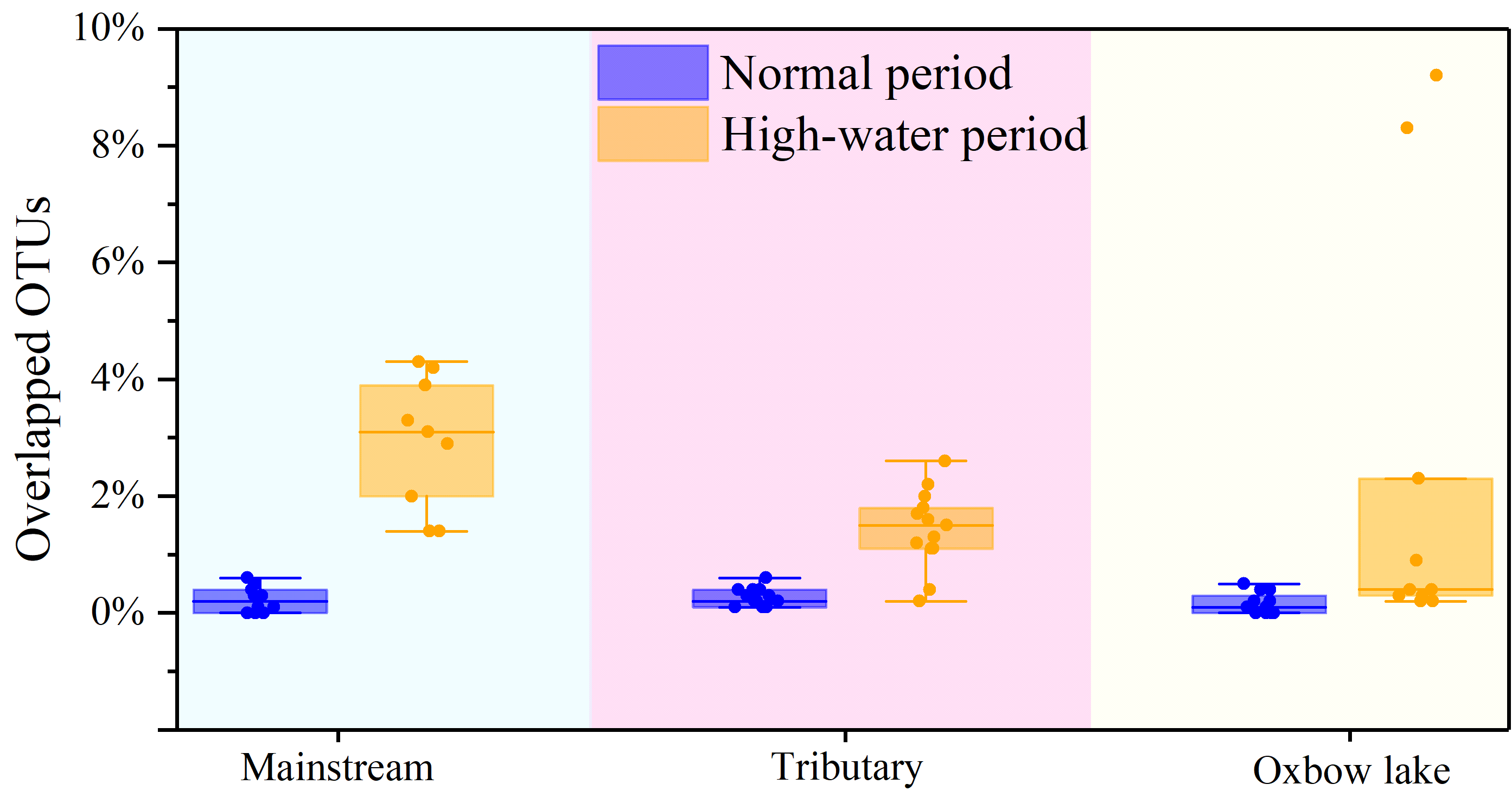


**Fig. S7** Proportions of overlapping operational taxonomic units (OTUs) between water and sediment samples at each sampling site relative to total OTUs for both categories.


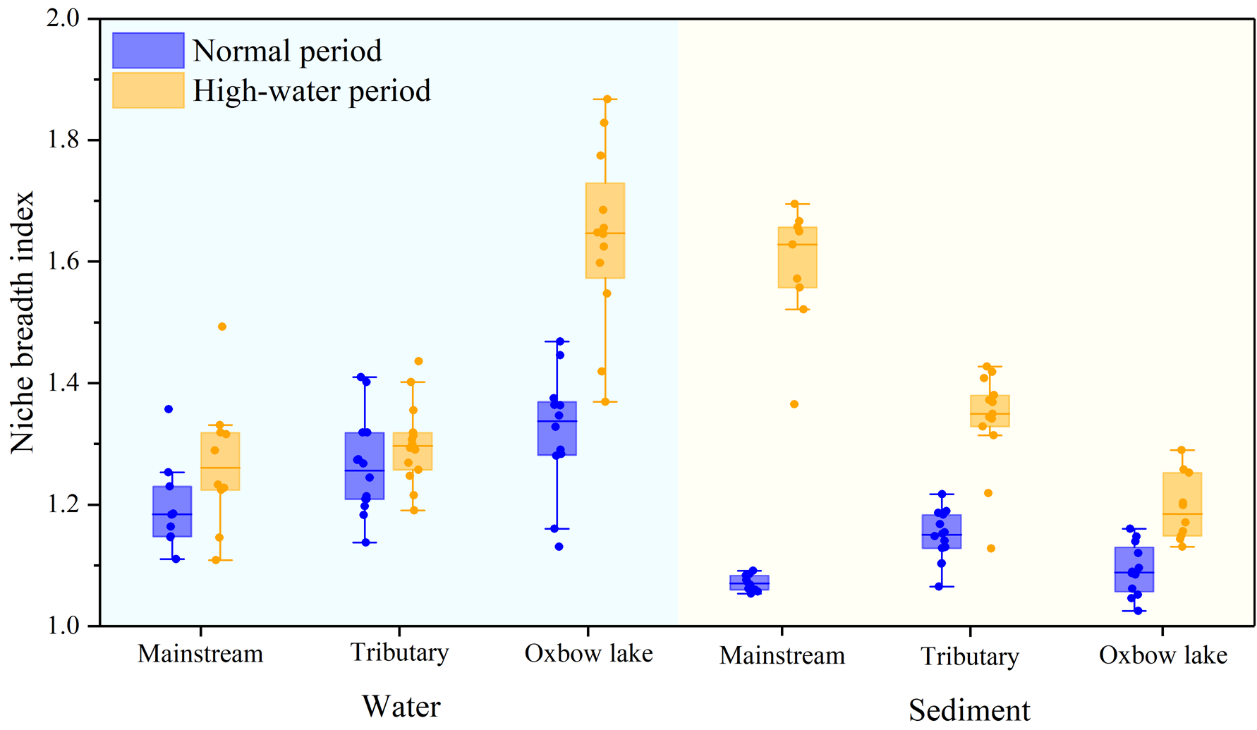


**Fig. S8** Comparison of mean habitat niche breadth of planktonic and sedimentary bacterial communities in the mainstream, tributaries and oxbow lakes in different periods.


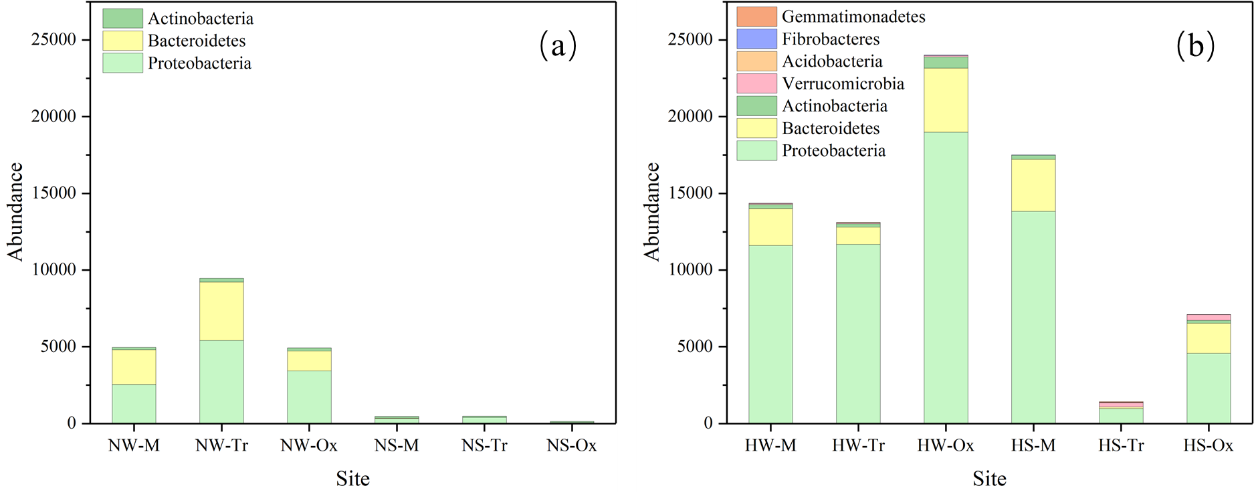


**Fig. S9** Abundances of keystone taxa in bacterial communities at the phylum level for six sub-networks. (a) Normal period. (b) High-water period
